# Supplementary material for: Facile Fabrication of α-Alumina Hollow Fiber-Supported ZIF-8 Membrane Module and Impurity Effects on Propylene Separation Performance
Source: Membranes (Basel). 2022 Oct 19;12(10):1015. doi: 10.3390/membranes12101015 (PMC9609772; doi:10.3390/membranes12101015)
Supplement: Supplementary file 1 [file membranes-12-01015-s001.zip › membranes-1969738-supplementary.pdf]

membranes

MDPI

Supplementary Materials

Facile Fabrication of α-Alumina Hollow Fiber-Supported ZIF-8 Membrane Module and Impurity Effects on Propylene Separation Performance

Taewhan Kim, Yeong Jae Kim, Chanjong Yu, Jongbum Kim and Kiwon Eum *

School of Chemical Engineering, Soongsil University, Seoul 06978, Korea; xoghks@soongsil.ac.kr (T.K.); a950625@soongsil.ac.kr (Y.J.K.); ych96@soongsil.ac.kr (C.Y.); bumparty95@soongsil.ac.kr (J.K.)
* Correspondence: kiwon.eum@ssu.ac.kr

Table S1. Summary of dry-jet-wet spinning condition for α-alumina hollow fiber fabrication.

**Dope Composition (PES/NMP/Al_2O_3/PVP) (wt %)**	6.8/38.0/54.7/0.5
**Dope Flow Rate (mL/h)**	120
**Bore Fluid**	DI-water
**Bore Fluid Flow Rate (mL/h)**	80
**Air Gap (cm)**	3
**Take up Rate (m/min)**	/*
**Operating Temperature (K)**	298
**Quench Bath Temperature (K)**	298

* Instead of using the take-up drum, the raw alumina fibers were collected from the bottom of the quenching bath.

Gas permeation test

The permeance of gas through the membrane, F_i, is defined as:

$$F_i = \frac{R_i}{\Delta P_i \times A} \quad \text{(S1)}$$

Where, R_i is the mole rate of component $i\ (mol/s)$, ΔP_i is the partial pressure difference component $i\ (Pa)$, across the membrane, and A is Area of the hollow fiber outer surface inside the membrane module (m^2).

When the feed is two or three gas mixture and the sweep gas flows inside the fiber, the concentration of components $i\ (ppm)$ passing through the membrane can be expressed as:

$$C_i = \frac{R_i}{R_0 + R_A + R_B} \quad (The\ feed\ is\ a\ mixture\ of\ two\ gases) \quad \text{(S2)}$$

$$C_i = \frac{R_i}{R_0 + R_A + R_B + R_C} \quad (The\ feed\ is\ a\ mixture\ of\ three\ gases) \quad \text{(S3)}$$

R_0 is the mole rate of sweep gas (mol/s), which can be obtained from the ideal gas equation ($\Delta P_0 V_0 = R_0 RT$), where ΔP_0, V_0, R, T means the partial pressure, volumetric flow rate, gas constant, and temperature of the sweep gas, respectively.

Since the C_i value can be found by plotting the Gas Chromatography results, the R_i value can be obtained using the above equation.

$$R_i = \frac{R_0 \times C_i}{1 - C_A - C_B} \quad (The\ feed\ is\ a\ mixture\ of\ two\ gases)) \tag{S4}$$

$$R_i = \frac{R_0 \times C_i}{1 - C_A - C_B - C_C} \quad (The\ feed\ is\ a\ mixture\ of\ three\ gases) \tag{S5}$$

The mole flow rate before passing through the membrane can be calculated by the following equation

$$R_{0,i} = \frac{P_F \times V_i}{R \times T} \tag{S6}$$

Where, P_F is the feed pressure (pa), V_i is the initial volumetric flow rate of the component i (m^3/s).

By Dalton's law of partial pressure, partial pressure difference component i (ΔP_i) can be calculated as follows

$$\Delta P_i = P_F \times \frac{R_{0,i}}{R_{0,A} + R_{0,B}} - P_S \times C_i \quad (binary\ gas\ mixture) \tag{S7}$$

$$\Delta P_i = P_F \times \frac{R_{0,i}}{R_{0,A} + R_{0,B} + R_{0,C}} - P_S \times C_i \quad (tertiary\ gas\ mixture) \tag{S8}$$

Where, P_S is the pressure of the sweep gas (pa).

The permeability of gas through the membrane, P_i, is defined as:

$$P_i = F_i \times l \tag{S9}$$

Where, l is the thickness of membrane (m).

The selectivity ($\alpha_{i,j}$) for component i over component j is defined as:

$$\alpha_{i,j} = \frac{P_i}{P_j} \tag{S10}$$

Where, P_i and P_j are the permeability of component i and j ($\frac{mol \cdot m}{m^2 \cdot pa \cdot s}$).

The separation factor ($\alpha_{i,j}$) for component i over component j is defined as:

$$\alpha_{i,j} = \frac{y_i/y_j}{x_i/x_j} \tag{S11}$$

Where, x_i and x_j are the molar fraction of component i and j in feed stream, respectively, and y_i, y_j are the molar fraction of components i and j in the permeate stream, respectively.

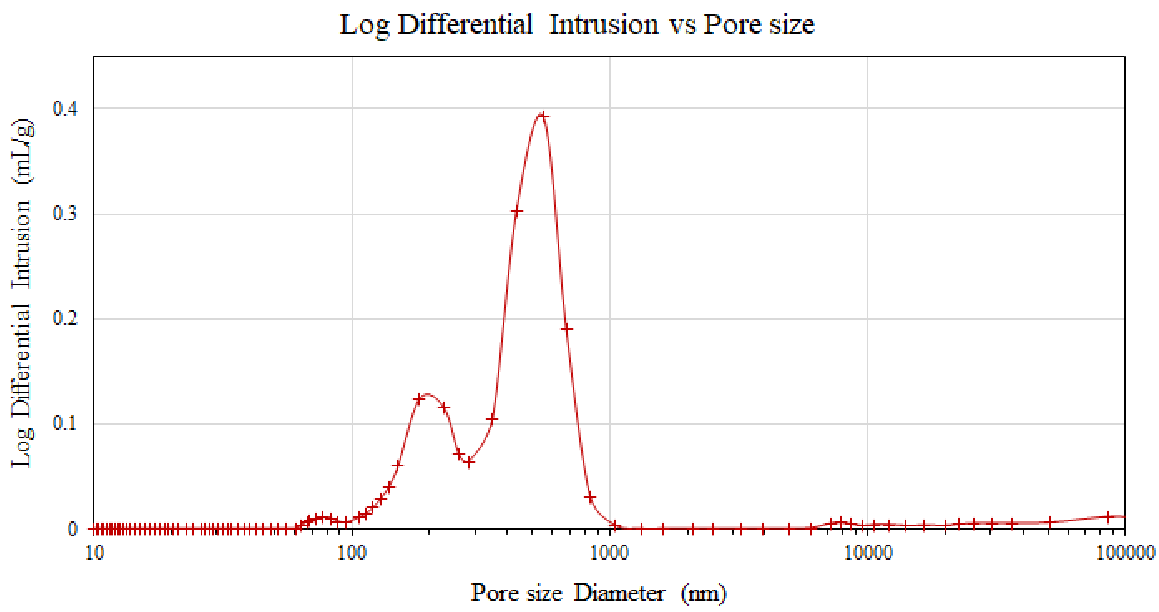

Figure S1. Pore size distribution of alumina hollow fiber by mercury porosimetry.
